# A comparison of time-of-flight mr angiography, contrast-enhanced mr angiography and ct angiography to evaluate vessel area in a rabbit peripheral arterial disease model

**DOI:** 10.1186/1532-429X-13-S1-P366

**Published:** 2011-02-02

**Authors:** Yi Xu, Y Fu, D Kedziorek, N Azene, T Ehtiati, A Flamang, SM Shea, DL Kraitchman

**Affiliations:** 1 Johns Hopkins Universtiy, Baltimore, MD, USA

## Introduction

Inducing arteriogenesis using stem cells is a promising treatment for patients suffering from peripheral arterial disease (PAD). Computed tomography angiography (CTA) is often used as a gold standard method for evaluating vessel diameters in ischemic tissue after therapy.

## Purpose

The purpose of our study was to compare the vessel area measurement in Time-of-Flight (TOF) MR angiography (MRA), contrast-enhanced MRA (ceMRA) and CTA after transplantation of mesenchymal stem cells (MSCs) in an animal PAD model.

## Methods

**Figure 1:**
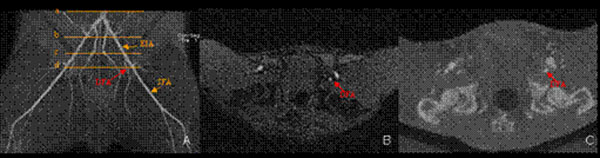
A maximum intensity projection TOF image (A) shows the vessel areas measurement at different level. The four orange lines (a-d) denote the position located at distal aorta (a), the proximal (b) and distal external iliac artery (EIA) (c), and the proximal deep femoral artery (DFA) (d), respectively. The left DFA (red arrow) is demonstrated at an axial image on TOF MRA and CTA (B-C).

Thirteen female New Zealand White rabbits were randomized to receive either intramuscular sham injection (n=4), empty capsules (n=4) or microencapsulated MSCs (n=5) in the medial thigh 24h after endovascular occlusion of the left superficial femoral artery (SFA). Three-dimensional (3D) TOF MRA acquired in the axial plane (3T Trio Siemens, 30ms TR; 3.69ms TE; 0.6×0.4×0.6mm^3^ voxel size), 3D ceMRA acquired in the coronal plane (3T Trio Siemens, 4.27ms TR; 1.73ms TE; 0.4×0.4×0.8mm^3^ voxel size; 15ml Gadobenate Dimeglumine 185mg/mL with flow rate 0.1mL/s ) and a C-arm CTA (Axiom Artis dFA, Siemens, 8s rotation; 240° scan angle; 0.37×0.37×0.37mm^3^ voxel size;16 ml Iohexol at 4ml/s IV) were acquired in random order prior to SFA occlusion, and 7 and 14 days after occlusion. Vessel areas at seven locations were measured (Figure 1) using custom software (VesselMass, The Netherlands). A reproducibility analysis and the correlation between CTA and TOF-MRA methods were performed. Values are reported as mean ± standard deviation.

## Results

**Figure 2:**
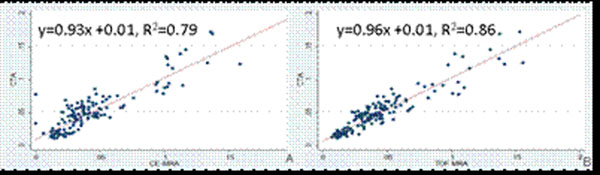
The correlation of vessel area measurement between TOF MRA and CTA (A, r2=0.86) showed a higher agreement than between ceMRA and CTA (B, r2-0.79).

Vessel areas in the aorta were 0.116 ± 0.02cm^2^ and decreased to on average 0.012 ± 0.003cm^2^ in the distal deep femoral artery. Both ceMRA and TOF MRA showed a high agreement with CTA with slightly better agreement by TOF MRA than ceMRA (r^2^=0.86 vs. r^2^=0.79, Figure 2). Intra-observer agreement for vessel areas detection was excellent by CTA and TOF MRA (r^2^=0.99) images.

## Conclusions

Measurement of small vessel diameters by MRA is challenging especially in ischemic or atherosclerotic disease. TOF MRA is traditionally considered a poor method for measuring vessel size in areas of slow flow. However, agreement of TOF MRA with CTA was higher than ceMRA in our rabbit PAD model. Potential degradation of ceMRA image resolution occurred due to reformatting of the ceMRA in the axial plane for measurement. However, both techniques showed an acceptable precision to measure small vessel diameters without ionizing radiation for the assessment of efficacy of an arteriogenesis therapy.

